# IgG4‐related lymphadenopathy: Benign but with rare recurrent

**DOI:** 10.1002/jha2.327

**Published:** 2021-10-30

**Authors:** Kevin Song, Qin Huang

**Affiliations:** ^1^ Department of Pathology and Laboratory Medicine Cedars‐Sinai Medical Center Los Angeles California USA

A 43‐year‐old Hispanic healthy male presented in 2018 with new onset of isolated left axillary lymphadenopathy with no fever, night sweats, weight loss, or other B‐symptoms. Outside hospital needle core biopsy reported conflicting pathology with concern for small lymphocytic lymphoma versus atypical lymphoid hyperplasia. A subsequent excisional biopsy was performed and showed an enlarged lymph node (2.4 cm) with nodular effacement of normal lymph node architecture with marked follicular hyperplasia and focal progressive transformation of germinal centers. Most of the germinal centers demonstrated intrafollicular plasmacytosis with scattered immunoblasts and eosinophils, and focal karyorrhectic debris with fibrin deposition (Figure 1, panels A and B). Follicular dendritic meshwork was intact. No interfollicular expansion and significant fibrosis were seen. Germinal center cells were positive for CD20, CD10, and BCL6 but negative for BCL2 by immunohistochemistry. Most of the plasma cells were positive for IgG and IgG4 (Figure 1, panels C and D), with an increased IgG4 versus IgG ratio up to 80%–90% and had greater than 100 IgG4 positive plasma cells per high power field. No light chain restriction was identified. Serum IgG4 level was slightly elevated at 94.8 mg/dl. Human herpesvirus‐8 and Epstein‐Barr Virus (EBV) were negative. The overall clinical and pathologic findings were consistent with IgG4‐related lymphadenopathy. The patient was followed with uneventful and no specific treatment after the procedure till October 2020, when he developed an isolated lymphadenopathy (3–4 cm) again at the exact same left axillary region with uncomfortable feeling and lateral pain over last 2–3 months. Physical examination and laboratory studies at the time were unremarkable. Given the recurrent nature of the lesion and clinical concerns for lymphoma, the patient underwent another excisional biopsy. The pathologic examination revealed almost identical morphologic and immunophenotypic features as the excisional biopsy 2 years prior, consistent with a recurrent disease. Further work‐up with immunoglobulin receptor gene rearrangement by PCR analysis and t(14;18) translocation by Fluorescence In Situ Hybridization (FISH) analysis were completely negative, supporting the diagnosis.

**FIGURE 1:**
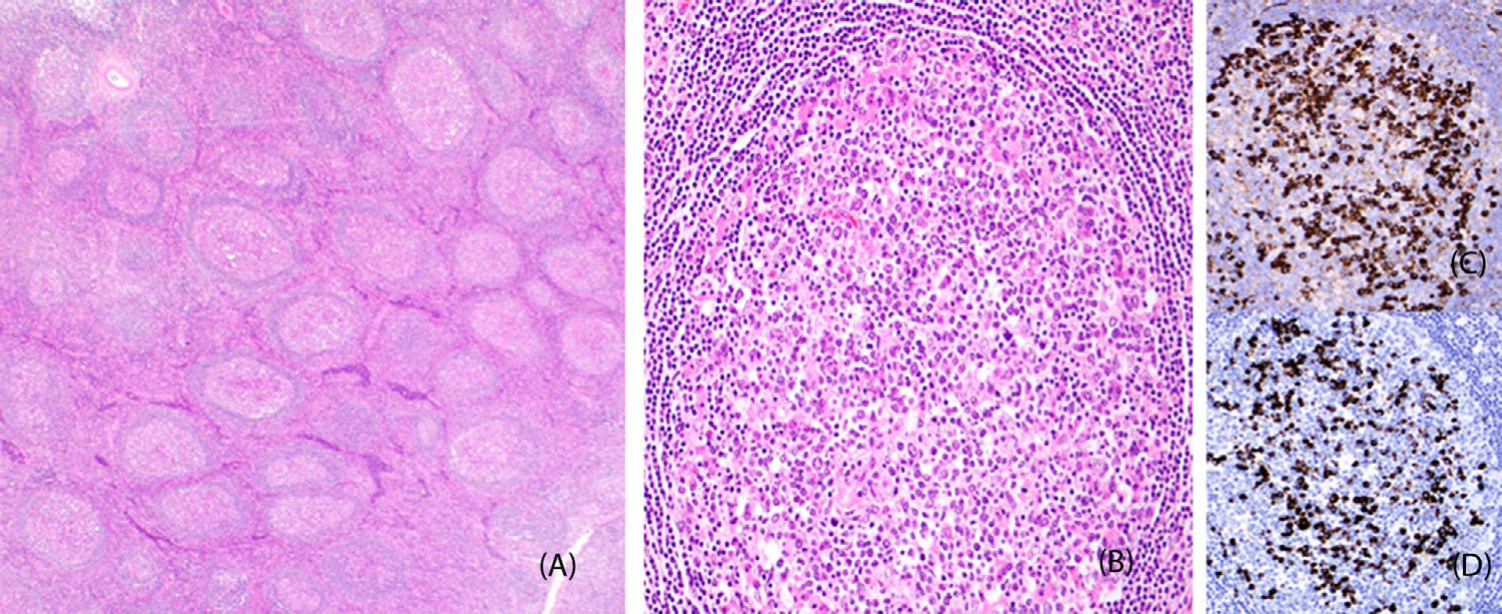
Lymph node with marked follicular hyperplasia and intrafollicular plasmacytosis (panels A and B, hematoxylin and eosin stain, original magnifications 100× and 400×, respectively). Intrafollicular plasmacytosis with increased IgG (panel C) versus IgG4 (panel D) ratio (immunohistochemistry, original magnifications, 400×)

IgG4 disease is a fibroinflammatory condition characterized by tumefective, sclerosing lesions involving virtually every organ system with diverse clinical manifestations. IgG4‐related lymphadenopathy (a newly recognized) can be the primary presentation of IgG4‐related disease. It may be localized or generalized and is usually associated with extranodal lesions [1, 2]. While the exact etiology and pathogenesis remain unclear, many patients with IgG4‐related lymphadenopathy respond well with steroid and anti‐CD20 therapies. However, recurrence may rarely occur, as the current case.

## CONFLICT OF INTEREST

The authors declare no conflict of interest.

## AUTHOR CONTRIBUTIONS

Both authors contributed to the paper.
